# Structural and functional characterization of CREB-binding protein (CREBBP) as a histone propionyltransferase

**DOI:** 10.1016/j.jbc.2025.110444

**Published:** 2025-07-02

**Authors:** Guiling Cui, Marie Ley, Ariel E. Mechaly, Linh-Chi Bui, Christina Michail, Jérémy Berthelet, Julien Dairou, Haopeng Yang, Guillaume Chevreux, Gautier Moroy, Michael R. Green, Ahmed Haouz, Fernando Rodrigues Lima

**Affiliations:** 1 Université Paris Cité, CNRS, Unité de Biologie Fonctionnelle et Adaptative, Paris, France; 2 Université Paris Cité, CNRS, Institut Jacques Monod, Plateforme ProtéoSeine, Paris, France; 3 Institut Pasteur, CNRS, Plateforme de Cristallographie-C2RT, Paris, France; 4 Université Paris Cité, CNRS, Unité Epigénétique et Destin Cellulaire, Paris, France; 5 Université Paris Cité, CNRS, Laboratoire de Chimie et de Biochimie Pharmacologiques et Toxicologiques, Paris, France; 6 Department of Lymphoma and Myeloma and Department of Genomic Medicine, The University of Texas MD Anderson Cancer Center, Houston, Texas, USA; 7 Université Paris Cité, INSERM, Unité de Biologie Fonctionnelle et Adaptative, Paris, France

**Keywords:** lysine propionylation, acetyltransferase, protein acylation, propionyl-CoA, crystal structure, CRISPR–Cas9, CREBBP

## Abstract

In addition to histone acetylation, histone lysine propionylation (such as the H3K18Pr mark) has recently attracted significant attention as a common and abundant modification linking the cellular metabolic state and gene expression. CREB-binding protein (CREBBP) and EP300 are key histone acetyltransferases that play a critical role in gene expression through their catalytic activity. Although CREBBP and EP300 are homologous enzymes with high structural similarities, they exhibit both redundant and specific functions. Dissecting the shared and divergent properties of CREBBP and EP300 is thus important to understand their roles. However, despite the importance of CREBBP, most mechanistic and structural studies have focused on EP300, leaving much less information about CREBBP. Interestingly, recent enzymatic and structural studies have demonstrated that EP300 can also function as a histone propionyltransferase. Using a combination of acyltransferase assays with different acyl-CoA cofactors and with peptides, recombinant histone, or recombinant nucleosomes as substrates, we provide enzymatic evidence that, in addition to its well-documented acetyltransferase activity, CREBBP readily propionylates histone H3 *in vitro*, notably generating the H3K18Pr mark. Importantly, subsequent cellular studies using CRISPR–Cas9-edited cells further support that CREBBP can act as a histone propionyltransferase *in vivo*, notably depositing the H3K18Pr mark. Finally, the crystal structures of the human CREBBP histone acetyltransferase domain in complex with propionyl-CoA or in complex with Lys-CoA provide the structural basis for the histone propionyltransferase properties of CREBBP. Taken together, these findings provide new insights into the enzymatic functions of CREBBP and a better understanding of the mechanisms linking cellular metabolism and epigenetic regulation.

Histones are modified by a variety of post-translational modifications that mediate a critical interplay between epigenetic regulation and metabolism (1). In addition to canonical lysine acetylation, other short-chain lysine acylations have been identified on histones, including propionylation, butyrylation, and crotonylation. The presence of these modifications on histones is determined by the cellular metabolic state and the availability of different forms of acyl-CoA (2). Notably, most of these histone acylation sites overlap with known histone acetylation sites and are catalyzed by histone acetyltransferases (HATs) (1, 3, 4). Among histone acylations, histone lysine propionylation has been shown recently to be a common and an abundant modification (5). Furthermore, histone lysine propionylation has been shown to be an active chromatin mark that, in combination with acetylation, links cellular metabolic state to chromatin structure and transcription (6, 7, 8). Recent studies have also shown that histone propionylation, in particular the H3K18 propionylation mark (H3K18Pr), is widely localized in the genome of colorectal cells and that this genomic localization is altered in colorectal cancer (9). It has been reported that propionyl-CoA (Pr-CoA) is significantly enriched in the nucleus of cells and thus contributes to H3 propionylation, particularly on H3K18 to generate the H3K18Pr epigenetic mark (10). Increased levels of Pr-CoA and protein lysine propionylation have also been reported in propionic acidemia, a metabolic disorder caused by deficiency of Pr-CoA carboxylase (11).

Recent studies have shown that major families of HATs can catalyze the acylation of histones and nonhistone proteins *in vitro* using acyl-CoA cofactors (4, 7). Although the preference for competing acyl-CoA cofactors depends mainly on the size of the acyl donor chain (with shorter chains generally being better cofactors), lysine acylation can be induced in a dose-dependent manner in response to increasing levels of given acyl-CoAs. In addition, cellular levels of acyl-CoAs, particularly Pr-CoA, span orders of magnitude and are correlated with the relative abundances of histone acyl marks *in vivo* (4, 7, 10).

The HAT CREB-binding protein (CREBBP or CBP) and its paralog EP300 (p300) play critical roles in chromatin structure and gene expression involved in numerous cellular processes (12, 13, 14, 15). Dysregulation of their catalytic activities is associated with several diseases, including lymphoma, leukemia, and Rubinstein–Taybi syndrome (16, 17, 18, 19, 20). CREBBP and EP300 exert their regulatory functions by catalyzing the acetylation of lysine residues on histones (particularly H3K18 and H3K27) and transcription factors such as p53 (14, 15, 21). Despite being homologs with high sequence and structural similarity, several studies indicate that CREBBP and EP300 exhibit both redundant and unique functions, as evidenced by the inability of EP300 to fully compensate for the loss of CREBBP and *vice versa* (21, 22, 23, 24, 25). For instance, it has been shown that the PHD finger domain of EP300, but not that of CREBBP, is dispensable for enzymatic activity (26). Moreover, despite their nearly identical sequence and structure, the KIX domains of EP300 and CREBBP display selective binding to transcription factor binding (27, 28). In addition, CREBBP and EP300 may differ in their specificity and selectivity toward substrates (21, 23). Therefore, identifying and characterizing the shared and unique properties of CREBBP and EP300 is of prime importance in order to better understand the roles of these enzymes. However, as most studies of the structure and function of CREBBP and EP300 paralogs have focused on EP300, much less information is available for CREBBP (21).

Previous work at both the enzymatic and structural levels has shown that EP300, in addition to its HAT properties, could also act as a histone propionyltransferase (29). In this study, we provide molecular, cellular, and structural insights showing that, similarly to EP300, CREBBP catalyzes lysine propionylation on H3 (generating notably the H3K18Pr mark) *in vitro* and *in vivo*. In addition, CREBBP performs both autopropionylation and propionylation of p53. The crystal structure of the HAT domain of human CREBBP in complex with Pr-CoA reveals a similar mode of binding of the cofactor to EP300, further providing mechanistic and structural evidence that CREBBP, in addition to its well-known acetyltransferase activity, can utilize Pr-CoA to deposit propionylation marks. The results reported in this study offer novel insights into the enzymatic function of CREBBP and support a broader physiological impact of this enzyme. In light of the mounting importance of histone propionylation in the crosstalk between cellular metabolism, chromatin structure, and gene expression, further studies are warranted to better understand the role of CREBBP-dependent propionylation (9, 10).

## Results and discussion

**Figure 1:**
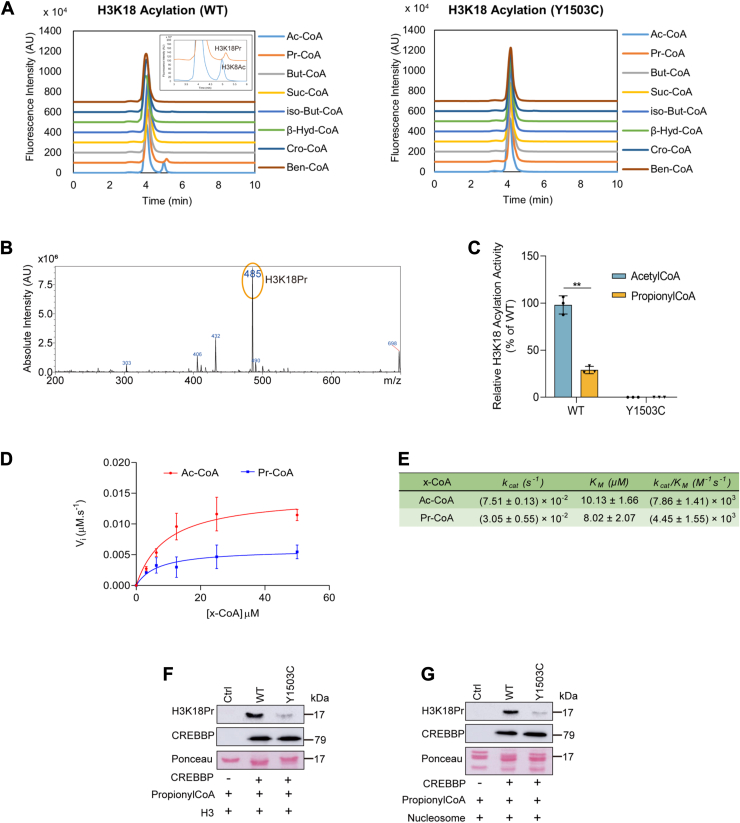
**Acyltransferase activity of recombinant human CREBBP.***A*–*C,* electrospray ionization (ESI) LC–MS analysis of H3K18 peptide acylation by recombinant CREBBP WT (catalytic core) or the inactive CREBBP Y1503C mutant. *A,* chromatograms showing H3K18 acylation by CREBBP (*left panel*) or CREBBP Y1503C mutant (*right panel*) in the presence of different acyl-CoAs. *B,* LC–MS spectrum showing H3K18 propionylation by CREBBP in the presence of Pr-CoA (*m/z* ratio equal to 485 Da, corresponding to the triple charged ion calculated as ([M + 3H^+^])/3). The molecular mass of the H3K18Pr peptide is 1453.61 Da. *C,* quantification of H3K18 acetylation and propionylation by CREBBP and CREBBP Y1503C. Experiments were performed in three biological replicates. *Bar graphs* and *error bars* represent the mean and SD. ∗∗*p* < 0.01. *D,* Michaelis–Menten saturation curves for Ac-CoA or Pr-CoA acylation of the H3K18 peptide substrate by CREBBP. Experiments were performed in three biological replicates, and error bars represent SD. *E,* kinetic parameters of Ac-CoA or Pr-CoA for CREBBP. Data are mean ± SD. *k*_cat_ and *k*_cat_/*K*_*m*_ values were significantly different (*p* < 0.05). *F,* western blot analysis of H3K18 propionylation of recombinant histone H3 by CREBBP or CREBBP Y1503C mutant. Recombinant CREBBP is detected with anti-6xHis tag antibody. Ponceau staining reveals recombinant histone H3. *G,* western blot analysis of H3K18 propionylation of recombinant nucleosomes by CREBBP or CREBBP Y1503C mutant. Recombinant CREBBP is detected with anti-6xHis tag antibody. Ponceau staining reveals the recombinant nucleosomes. Ac-CoA, acetyl-CoA; CREBBP, CREB-binding protein; H3K18Pr, H3K18 propionylation mark; Pr-CoA, propionyl-CoA.

**Figure 2:**
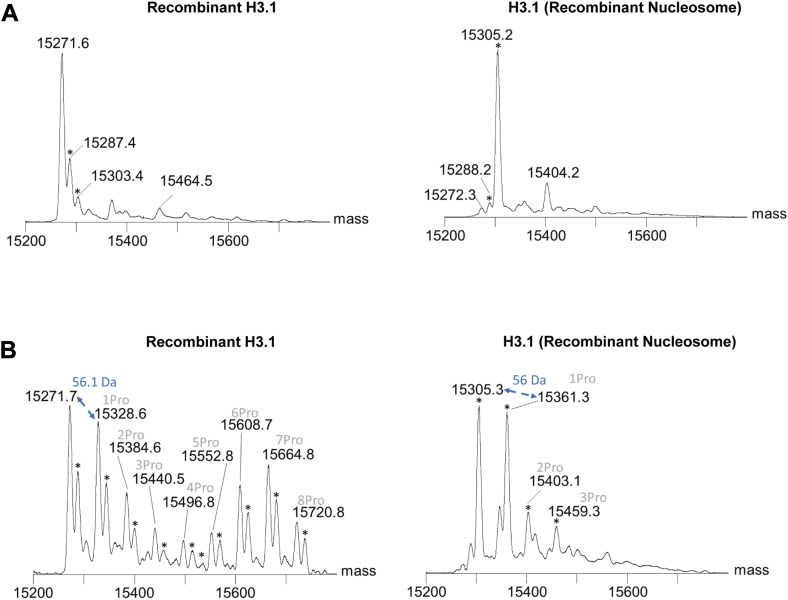
**Mass spectrometry analysis of intact recombinant histone H3 and recombinant nucleosomes (histone H3) after incubation with Pr-CoA in the presence or the absence of CREBBP**. *A,* mass spectra of intact recombinant H3 (*left panel*) and recombinant H3 in nucleosomes (*right panel*) incubated with Pr-CoA. Molecular mass (in atomic mass units) and methionine oxidation (∗) are indicated. *B,* mass spectra of intact recombinant H3 (*left panel*) and recombinant H3 in nucleosomes (*right panel*) incubated with Pr-CoA and CREBBP. Molecular mass and number of propionylation events are shown. Methionine oxidation (∗) is indicated. CREBBP, CREB-binding protein; Pr-CoA, propionyl-CoA.

Among the different histone H3 and H4 lysines that can be acetylated by CREBBP, the enzyme displays a high specificity for H3K18 (and to a lesser extent for H3K27) *in vitro* and *in vivo* (23, 30, 31). To evaluate the acyltransferase activity of purified recombinant human CREBBP (catalytic core, residues: 1095–1773; Fig. S1*A*), *in vitro* assays based on an H3K18 peptide substrate, different acyl-CoAs and ultrafast liquid chromatography (UFLC) coupled to fluorescence and mass spectrometry (MS) detection were performed as previously reported (32, 33). As shown in Figure 1*A* (*left panel*), only acetylation and propionylation of the H3K18 peptide substrate by CREBBP could be readily detected by fluorescence-based UFLC. As previously reported for EP300, we did not observe any significant acyltransferase activity with acyl-CoAs with longer acyl chains further supporting recent studies suggesting that CREBBP, like EP300, may act primarily as an acetyl- and/or propionyltransferases using acetyl-CoA (Ac-CoA) and Pr-CoA as cofactors (Fig. 1*A*) (4, 29). Neither acetylation nor propionylation of the H3K18 peptide was observed with a catalytically inactive mutant of CREBBP (CREBBP Y1503C), confirming that the K18 modifications are dependent on the enzymatic activity of CREBBP (Fig. 1*A*, *right panel*). Propionylation and acetylation of K18 on the H3 peptide substrate were further confirmed by UFLC–MS (with *m/z* values for the M + 3H^+^ ion of 485 and 481 for propionylation and acetylation of the H3K18 peptide, respectively) (Figs. 1*B* and Fig. S1*B*). Furthermore, both modifications were also observed with H3K27 and p53K382 peptide substrates, consistent with the fact that these lysine residues in H3 and p53 proteins are known lysine targets of CREBBP (Fig. S2) (21). As previously shown for EP300, the propionyltransferase activity of CREBBP on H3 peptide substrate *in vitro* was found to be approximately 33% of the acetyltransferase activity of the enzyme (Fig. 1*C*) (29). Further kinetic analysis indeed confirmed that the catalytic efficiency (*k*_cat_/*K*_*m*_) of CREBBP for propionylation of the H3K18 peptide substrate is twofold lower than for acetylation (*k*_cat_/*K*_*m*_ = 4.45 ± 1.55 × 10^3^ M^-1^s^-1^ and *k*_cat_/*K*_*m*_ = 7.86 ± 1.41 × 10^3^ M^-1^s^-1^ for propionylation and acetylation, respectively) (Fig. 1, D and E). The *K*_*m*_ values of Pr-CoA and Ac-CoA for CREBBP were equal to 8.02 ± 2.07 μM and 10.13 ± 1.66 μM, respectively. This suggests that the enzyme binds to the two cofactors with similar affinity but displays a higher catalytic turnover number of Ac-CoA. Of note, Henry *et al.* (23) reported similar steady-state kinetic parameters for H3K18 acetylation by CREBBP and EP300, with catalytic efficiencies (*k*_cat_/*K*_*m*_) ranging from 4 to 14 × 10^3^ M^-1^s^-1^ and, notably, a *K*_*m*_ value for Ac-CoA ranging from 2 to 7 μM. Analogous results showing similar affinity (*K*_*m*_ = 1 μM) for Ac-CoA and Pr-CoA, and higher *k*_cat_ for Ac-CoA were reported for the HAT MOF (males absent on the first) (34). It is known that the abundance of Ac-CoA and Pr-CoA in cells varies depending on their metabolic state and/or altered activities of metabolic enzymes and can reach concentrations well above 20 μM, thus supporting that CREBBP, like EP300, would be able to act as both an acetyl- and/or propionyltransferase *in vivo* (4, 9, 10, 11, 35). To further confirm the histone H3 propionyltransferase properties of CREBBP, we performed Western blot assays with recombinant purified human histone H3 or nucleosomes using a commercial anti-H3K18 propionylation (anti-H3k18Pr) antibody, which has been used in recent studies to characterize this epigenetic mark (H3K18Pr) in normal and cancerous human cells (9). Consistent with the results reported previously, we found that H3K18 was readily propionylated *in vitro* by CREBBP but not by the inactive form of the enzyme (CREBBP Y1503C), when purified recombinant histone H3 or nucleosomes were used as substrates (Fig. 1, *F* and *G*). Further LC–MS/MS experiments were performed with purified recombinant histone H3 or nucleosomes incubated with Pr-CoA in the presence or the absence of CREBBP enzyme. In agreement with the data reported previously, a series of mass peaks (+56.01 Da mass shift) corresponding to lysine propionylation events in histone H3 were observed (Fig. 2). Indeed, several histone H3 lysines known to be acetylated by CREBBP, in particular H3K18, were found to be propionylated by the enzyme (Figs. S3 and S4) (23). These results are consistent with previous MS analyses showing that both EP300 and CREBBP readily propionylate key H3 lysine residues, including H3K14, H3K18, H3K23, or H3K27 (4, 29, 36). In addition to histone H3, CREBBP is also known to acetylate nonhistone proteins, including p53, and to autoacetylate itself (13, 21). Western blot assays were performed using purified recombinant p53 or CREBBP (catalytic core) proteins and a commercial pan anti–propionyl-lysine antibody recently used to characterize protein lysine propionylation (4, 10). As expected, p53 and CREBBP were found to be propionylated by CREBBP, which is also consistent with previous studies (Fig. S5) (37).

**Figure 3:**
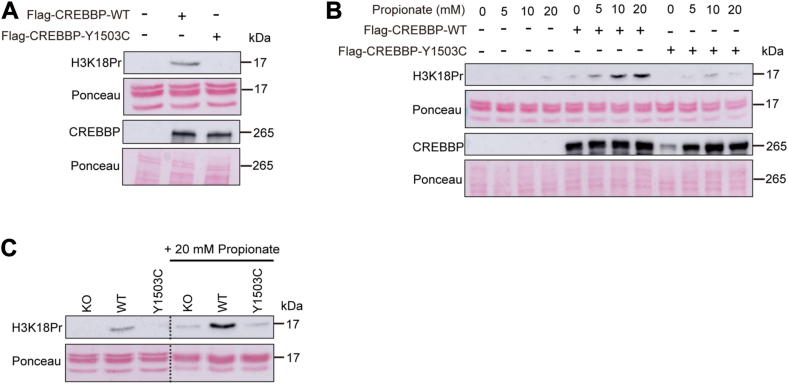
***In vivo* H3K18 propionylation (H3K18Pr) by CREBBP**. *A,* CRISPR CREBBP KO HEK293T cells were transfected for 48 h with a FLAG-tagged plasmid encoding human CREBBP WT or the inactive CREBBP Y1503C mutant. Deposition of the H3K18Pr mark on cellular histones and expression of FLAG-CREBBP enzymes in transfected cells were analyzed by Western blot using anti-H3K18Pr and anti-FLAG antibodies, respectively. Nontransfected cells were used as controls. Ponceau staining is shown. *B,* CRISPR CREBBP KO HEK293T cells were transfected with a FLAG-tagged plasmid encoding human CREBBP WT or the inactive mutant CREBBP Y1503C for 24 h. Cells were further grown in the absence or the presence of different concentrations of propionate prior to Western blot analysis as described. Nontransfected cells were used as controls. Ponceau staining is shown. *C,* CRISPR CREBBP KO RL cells, CRISPR CREBBP WT RL cells, and CRISPR CREBBP Y1503C RL cells were grown in the absence or the presence of 20 mM propionate prior to Western blot detection of the H3K18Pr mark. Ponceau staining is shown. CREBBP, CREB-binding protein; HEK293T, human embryonic kidney 293T cell line.

Taken together, the biochemical experiments reported previously indicate that recombinant CREBBP catalyzes histone H3 propionylation, specifically the H3K18Pr mark, *in vitro*. To analyze whether CREBBP can catalyze histone H3 lysine propionylation and specifically deposition of the H3K18Pr mark in a cellular context, we performed transfection studies in human embryonic kidney 293T (HEK293T) cells, as these cells have been used as a model to study endogenous histone lysine propionylation by different HAT enzymes such as EP300 or MOF (6, 34). As shown in Figure 3*A*, deposition of the H3K18Pr mark was observed in Western blots of endogenous histone H3 extracted from cells transfected with CREBBP. In contrast, no H3K18Pr mark was detected in cells transfected with the catalytically inactive form of CREBBP. The endogenous metabolite propionate is a precursor of Pr-CoA. Increased levels of propionate are known to lead to increased cellular levels of Pr-CoA and a subsequent increase in lysine propionylation, particularly in histones (9, 38). Consistent with this, we found that the H3K18Pr mark increased in CREBBP-transfected cells exposed to different physiological levels of propionate (Fig. 3*B*). Conversely, no significant H3K18Pr deposition was observed in cells transfected with the CREBBP Y1503C mutant and exposed to propionate (Fig. 3*B*). To further confirm that CREBBP can act as a histone propionyltransferase and generate the H3K18Pr mark *in vivo*, we used a model of isogenic CRISPR-edited human B-cell lymphoma cell lines (RL cell line), which were KO for CREBBP or expressed the WT or the catalytically inactive form (Y1503C) of the enzyme (20). RL cells harbor monoallelic frameshift mutations of the *EP300* gene, allowing for a less confounded assessment of the CREBBP properties (20). Consistent with the results reported previously, the H3K18Pr mark was observed in RL cells expressing CREBBP WT but not in cells expressing the inactive form of the enzyme (Fig. 3*C*). As expected, increased H3K18Pr deposition was observed in cells expressing CREBBP WT and exposed to propionate but not in KO or CREBBP Y1503C-expressing cells (Fig. 3*C*). Taken together, our results provide strong molecular and cellular evidence that CREBBP can catalyze histone lysine, particularly the H3K18Pr mark, *in vitro* and *in vivo*.

As mentioned previously, despite the biological importance of the CREBBP and EP300 enzymes, most studies have focused on EP300, leaving much less information about CREBBP (21, 29, 39, 40, 41, 42, 43). In addition, although CREBBP and EP300 are homologous HAT enzymes with a high degree of sequence identity, slight structural and functional differences may occur (21, 23, 44). Recent work has revealed the structure of the EP300 HAT domain bound to Pr-CoA (29). To elucidate the structural basis for the propionyltransferase activity of human CREBBP, we determined the structure of the HAT domain of the human enzyme in complex with Pr-CoA. To this end, we introduced the inactivating mutation Y1503F and deleted the flexible activity in the loop to favor protein crystallization, as previously shown for the apo-form of CREBBP (21).

**Figure 4:**
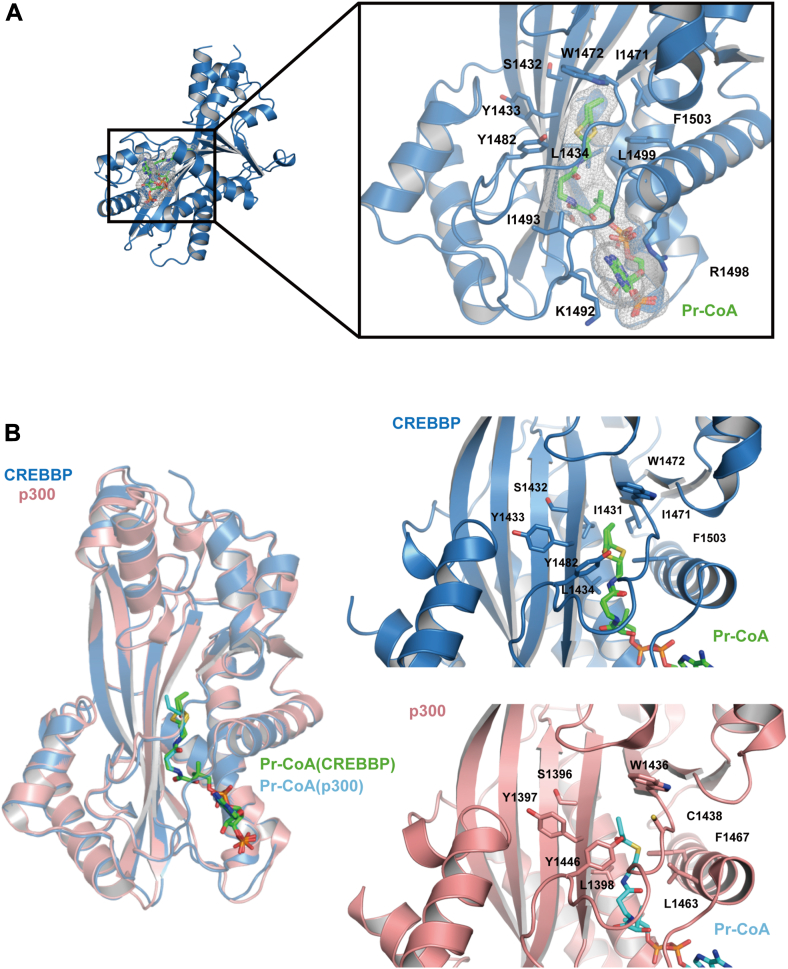
**Overall structure of the human CREBBP HAT domain in complex with Pr-CoA and comparison with the EP300–Pr-CoA complex.***A, ribbon* representation of the CREBBP HAT domain with bound Pr-CoA. Pr-CoA is shown in *sticks* and *mesh* (two conformers with 70% and 30% occupancy).The *inset* shows the main residues in the CREBBP HAT active site (*sticks*) that are in contact with Pr-CoA (in *sticks* and *mesh*). In particular, residues K1492 and R1498 are shown, which make contacts to the adenine ring of Pr-CoA in CREBBP (corresponding to K1456 and R1462 in EP300). *B,* structural alignment of EP300 (Protein Data Bank ID: 5LKX) and CREBBP HAT domains in complex with Pr-CoA (*left panel*). Close-up view of key residues in the CREBBP HAT active site (*upper right panel*) or EP300 HAT active site (*lower right panel*) that are in contact with the aliphatic pantothenic acid, β-mercaptoethylamine, and propionyl moieties of Pr-CoA: in particular, residues I1432, Y1433, L1434, W1472, and Y1482 in CREBBP (corresponding to residues I1395, Y1397, L1398, W1436, and Y1446 in EP300) are shown (*sticks*). CREBBP, CREB-binding protein; HAT, histone acetyltransferase; Pr-CoA, propionyl-CoA.

**Figure 5:**
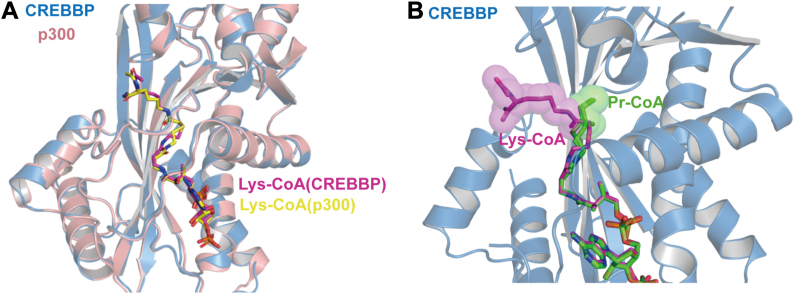
**Overall structure of human CREBBP HAT domain in complex with Lys-CoA and comparison with EP300–Pr-CoA complex or CREBBP–Pr-CoA complex**. *A,* structural alignment of the CREBBP and EP300 (Protein Data Bank ID: 3BIY) HAT domain in complex with Lys-CoA inhibitor (*left panel*). The Lys-CoA ligands are shown as *sticks*. Residues 1470 to 1488 and 1434 to 1452 in CREBBP and EP300 have been omitted for clarity. *B,* superposition of the CREBBP HAT–Lys-CoA and CREBBP HAT–Pr-CoA structures. The ligands are shown as *sticks*. The lysine moiety of Lys-CoA and the propionyl moiety of Pr-CoA are also shown in Van der Waals spheres. For clarity, only the HAT domain of the CREBBP–Pr-CoA structure is shown. Residues 1467 to 1486 have also been omitted for clarity. CREBBP, CREB-binding protein; HAT, histone acetyltransferase; Pr-CoA, propionyl-CoA.

The crystal structure of the human CREBBP HAT domain in complex with Pr-CoA was determined by molecular replacement using the CREBBP apo structure as a search model and refined to 1.7 Å (Fig. 4*A*) (Table 1) (21). The structure of the CREBBP HAT domain bound to Pr-CoA is similar to the apo form (backbone RMSD of 0.8 Å), consistent with previous studies suggesting that the cofactor binding site is stably formed in the absence of cofactor (21). The terminal thiol–propionyl moiety was found to adopt two slightly different conformations, refined to relative occupancies of 70% and 30% (Fig. 4, *A* and *B*). The overall structure of the CREBBP HAT domain with Pr-CoA is similar to that of the EP300 HAT domain with Pr-CoA (backbone RMSD of 0.5 Å) (Fig. 4*B*). The Pr-CoA molecules occupy the same positions within the cofactor binding pocket of the enzymes and display overall similar conformations with a slightly different conformation of the thiol–propionyl moieties (Fig. 4*B*). The cofactor binding pocket in the two liganded structures shares a common architecture in which Pr-CoA is accommodated by extensive interactions involving conserved residues (Fig. 4, *A* and *B*). For example, the adenosine ring of Pr-CoA is sandwiched by Arg1498 and Lys1492 in CREBBP, which correspond to residues Arg1462 and Lys1456 in EP300 (Fig. 4*A*) (24, 40). The aliphatic moiety of Pr-CoA is accommodated in a hydrophobic tunnel in both enzymes, where a number of conserved hydrophobic residues, in particular Tyr1482, Leu1434, Trp1472, Ile1431, or Tyr1433 (corresponding to residues Tyr1446, Leu1398, Trp1436, Ile1395, and Tyr1397 in human EP300, respectively) interacts with the pantothenic acid, β-mercaptoethylamine, and propionyl moieties of Pr-CoA, as previously observed in the EP300–Pr-CoA structure (Fig. 4*B*) (29, 41). As shown in the EP300–Pr-CoA structure, the Pr-CoA sulfur atom in the CREBBP HAT structure is directed toward the Phe1503 residue (corresponding to the catalytic Tyr1503 in CREBBP WT), which acts as a general acid during catalysis (Fig. 4*B*) (29, 41). Structural analyses of EP300 in complex with Pr-CoA and in complex with the inhibitor Lys-CoA suggest that the lower histone propionyltransferase activity of EP300 compared with its HAT activity is due to the fact that the extended propionyl moiety is accommodated in the lysine substrate-binding tunnel, thereby altering the substrate-assisted conformational rearrangement of the Pr-CoA chain with a subsequent decrease in the rate of catalysis (29). This mechanism would be even more applicable to acyl-CoA variants longer than Pr-CoA (such as butyryl-CoA [But-CoA]), which would be accommodated in the substrate-binding tunnel of EP300 in a conformation much less favorable for acyl transfer compared with acetyl transfer or, to a lesser extent, propionyl transfer (29). Determination of the structure of CREBBP HAT domain in complex with Lys-CoA at 2 Å shows that it is very similar to EP300–Lys-CoA complex (backbone RMSD of 0.4 Å) with the ligand adopting a similar position and conformation in both EP300 and CREBBP structures (Table 1 and Fig. 5*A*). The superposition of the Lys-CoA and Pr-CoA in CREBBP HAT structures clearly shows that the lysine moiety of Lys-CoA (mimicking the position of the lysine substrate) is mutually exclusive with the propionyl moiety of Pr-CoA, further supporting that the propionyl chain is accommodated in the lysine substrate tunnel, thereby leading to a less efficient catalytic rate, as observed for EP300 (Fig. 5*B*) (29).

**Table 1:** Data collection and refinement statistics

	CREBBP Pr-CoA (PDB ID: 9H0K)	CREBBP Lys-CoA (PDB ID: 9H02)
Wavelength	0.9801	0.9793
Resolution range (Å)	69.553–1.751 (1.930–1.751)	45.97–2.03 (2.11–2.03)
Space group	P 21 21 21	P 21 21 2
Unit cell (Å)	83.408 88.513 112.455 90 90 90	75.01 102.1 51.48 90 90 90
Total reflections	530,779 (23,810)	340,458 (34,828)
Unique reflections	58,454 (2923)	48,684 (5109)
Multiplicity	9.1 (8.1)	7.0 (6.8)
Completeness (%)	92.0 (56.4)	99.00 (93.83)
Mean I/sigma(I)	7.2 (1.6)	8.35 (1.53)
Wilson *B*-factor	19.30	33.06
*R*-merge	0.210 (2.003)	0.1696 (1.3)
*R*-meas	0.223 (2.138)	0.1835 (1.405)
*R*-pim	0.074 (0.741)	0.06943 (0.5273)
CC1/2	0.994 (0.616)	0.996 (0.58)
Reflections used in refinement	58,423 (83)	25,884 (2676)
Reflections used for *R*-free	3056 (4)	1294 (134)
*R*-work	0.1955 (0.4684)	0.1748 (0.2292)
*R*-free	0.2398 (0.5701)	0.2241 (0.3167)
Number of nonhydrogen atoms	5896	2932
Macromolecules	5246	2695
Ligands	213	24
Solvent	437	213
Protein residues	639	320
RMS (bonds)	0.007	0.008
RMS (angles)	0.99	1.03
Ramachandran favored (%)	98.42	97.46
Ramachandran allowed (%)	1.58	2.54
Ramachandran outliers (%)	0.00	0.00
Rotamer outliers (%)	1.04	2.07
Clashscore	4.28	5.38
Average *B*-factor	27.91	40.02
Macromolecules	27.24	39.77
Ligands	26.92	38.03
Solvent	36.54	43.88

Statistics for the highest-resolution shell are shown in parentheses.

## Conclusions

CREBBP and EP300 are homologous enzymes with high structural similarity that each display both redundant and unique functions. Identifying and characterizing their shared and divergent properties is therefore important for understanding the roles of CREBBP and EP300 (25). Recent enzymatic and structural studies have shown that, in addition to its HAT activity, EP300 can also propionylate histones (29). Interestingly, histone lysine propionylation (such as the H3K18Pr mark) has recently gained significant attention as a common and abundant modification linking the cellular metabolic state to gene expression (9). In this work, we conducted in-depth molecular and enzymatic studies demonstrating that CREBBP can also act as a histone and nonhistone propionyltransferase. Importantly, using a model of CRISPR–Cas9-edited human cells expressing an inactive form of CREBBP, we confirm that the enzyme can readily propionylate cellular histones *in vivo*, notably generating the H3K18Pr epigenetic mark. Finally, X-ray crystal structures of the HAT domain of human CREBBP in complex with Pr-CoA and Lys-CoA provide the structural basis for the propionyltransferase properties of CREBBP. Altogether, our results suggest that, similarly to EP300, CREBBP can function as a histone propionyltransferase. Our study further shows that CREBBP generates the activating epigenetic mark H3K18Pr *in vivo*, particularly in the presence of the Pr-CoA precursor propionate, thus further highlighting the link between cellular metabolism and epigenetic modifications.

## Experimental procedures

### Chemicals, reagents, and antibodies

Ac-CoA, Pr-CoA, But-CoA, DL-β-hydroxybutyryl-CoA, succinyl-CoA, iso-butyryl-CoA, crotonyl-CoA, ammonium bicarbonate, sodium propionate, Dulbecco's modified Eagle's medium, and RPMI were from Sigma. Benzoyl-CoA was from Santa Cruz Biotechnology. Recombinant human histone H3.1 was obtained from New England Biolabs. Recombinant human nucleosomes were from EpiCypher. MS grade acetonitrile (ACN), H_2_O, and formic acid (FA) were from ThermoFisher Scientific. Sequencing grade chymotrypsin was from Promega. Difluoroacetic acid was from Merck. Evotips Pure were from Evosep. Metafectene reagent was from Biontex. Anti-6xHis tag and anti-p53 antibodies were from Sigma. Anti-FLAG tag antibody was from Origene. Anti-CREBBP antibody was from Cell Signaling. Anti-H3K18Pr (PTM-213) and anti-pan-propionyl-lysine (PTM-201) were from PTM BIO, Inc. 5-FAM (5-carboxyfluorescein)-conjugated peptides were synthesized by Proteogenix: FAM-RAPRKQLAT-NH_2_ (H3K18 peptide), FAM-TKAARKSAPAT-NH_2_ (H3K27 peptide), and FAM-TSRHKKLMF-NH_2_ (p53).

### Protein expression and purification

The complementary DNA sequences of human CREBBP encoding the catalytic core of the enzyme (catalytic core: residues 1095–1773) or encoding the HAT domain with replacement of residues 1576 to 1580 by a 5-amino acid SGGSG linker and a Y1503C mutation (CREBBP HAT domain: residues 1322–1700) were cloned into pET28a expression vector and transformed into competent *Escherichia coli* BL21 codon plus. All constructs were confirmed by DNA sequencing. Bacteria were grown in yeast extract tryptone medium supplemented with 30 μg/ml kanamycin and 20 μg/ml chloramphenicol. IPTG (1 mM) was added to induce expression of the recombinant proteins when the absorbance value reached 0.6 to 0.7, followed by shaking overnight at 16 °C. Bacteria were harvested by centrifugation (4400*g*, 4 °C, 15 min) and resuspended in lysis buffer (20 mM Tris–HCl, 300 mM NaCl, 1% Triton X-100, 3 mM DTT, 1 mg/ml lysozyme, and 1x protease inhibitors, pH 8.0) for 30 min at 4 °C with agitation. The lysate was sonicated (10 s ON, 20 s OFF, 20% power) for 10 min, and the soluble fraction was clarified by centrifugation (15,000*g*, 4 °C, 30 min). The supernatant was then incubated with His-Select nickel affinity gel beads in the presence of 5 mM imidazole for 2 h at 4 °C. Beads were loaded on a column equilibrated with lysis buffer and washed with washing buffer (20 mM Tris–HCl, 300 mM NaCl, 1% Triton X-100, pH 8) followed by a second wash with washing buffer without Triton X-100. The proteins were eluted from the affinity chromatography column using 20 mM Tris–HCl, 300 mM NaCl, 300 mM imidazole, pH 8.0, followed by buffer exchange to 20 mM Tris–HCl, 150 mM NaCl, pH 8.0 using a desalting PD-10 column (Cytiva). P53 protein was purified from *E. coli* bacteria transformed with a pET-30 plasmid encoding human p53 as a 6XHis tagged protein (kind gift of Dr Hua LU, Tulane University). Protein concentration was measured using Bradford reagent, and protein purity was assessed by SDS-PAGE followed by Coomassie staining. Proteins were stored at −80 °C. For X-ray structure determination, the CREBBP HAT domain was further purified by size-exclusion chromatography on a Superdex column (Cytiva) and concentrated to 5 mg/ml.

### LC–MS analysis of the acylation of H3 and p53 peptide substrates by CREBBP

The CREBBP catalytic core (200 nM) was incubated with 250 μM acyl-CoAs and 100 μM H3K18 peptide in 50 μl assay buffer (25 mM Tris–HCl, pH 8.0) at room temperature for 20 min. The reaction was quenched by adding 50 μl of 15% HClO_4_, and 20 μl of the mixture was injected into the LC–MS system. Peptide and acyl-peptide products were separated on a Phenomenex C18 column and detected on a Shimadzu Nx8060 quadrupole ion analyzer equipped with an electrospray ionization probe operating at 250 °C. The separation was performed using an isocratic mobile phase (80% A/20% B), where A was water with 0.1% FA and B was ACN with 0.1% FA at a flow rate of 0.3 ml/min. The peptides and acyl peptide species were detected in selected ion monitoring mode with mass/charge ratios (*m/z*) of 1397.53 Da (H3K18 peptide), 1458.57 Da (H3K27 peptide), and 1504.73 Da (p53 peptide) for the native peptides; 1439.58 Da (H3K18Ac peptide), 1500.62 Da (H3K27Ac peptide), and 1546.78 Da (p53Ac peptide) for the acetyl peptides; and 1453.61 Da (H3K18Pr peptide), 1514.65 Da (H3K27Pr peptide), and 1560.81 Da (p53Pr peptide) for the propionyl peptides. Quantification was performed by integrating the peak areas employing a calibration curve established with various known concentrations of the two peptides as reported previously (32). For kinetic analysis of H3K18 peptide acetylation or propionylation by CREBBP, different concentrations of Ac-CoA or Pr-CoA (ranging from 0 to 50 μM) were used in the assay. Steady-state kinetic parameters were determined from nonlinear regression fitting of the data to the Michaelis–Menten equation using GraphPad Prism 8.0 (GraphPad Software, Inc) as done previously (32).

### Propionylation of recombinant protein substrates by CREBBP

CREBBP catalytic core (2 μg) was incubated with 100 μM Pr-CoA and 500 ng recombinant human H3.1 or 1 μg recombinant human nucleosome or 300 ng recombinant human p53 in 10 μl assay buffer (20 mM Tris–HCl, 1 mM DTT, pH 8.0) at room temperature for 20 min. Samples were analyzed by Western blot using anti-H3K18Pr or anti-pan propionyl lysine or anti-6XHis tag antibodies (see later). Samples were also analyzed by LC–MS and LC–MS/MS as described later.

### Western blotting

Samples were separated by SDS-PAGE followed by transfer to nitrocellulose membranes (0.45 μm; Cytiva). Membranes were blocked in 5% nonfat milk in PBS 0.1% Tween-20 (PBST) for 1 h and incubated with primary antibody in 1% nonfat milk PBST at 4 °C overnight. The next day, the membranes were washed three times with PBST before incubation with secondary antibody for 1 h at room temperature. The membranes were then washed, and the signal was detected by chemiluminescence using Enhanced Chemiluminescence Prime reagent on an Amersham Imager 600 detection system (Cytiva).

### LC–MS analysis of intact histone samples

Samples (corresponding to 200 ng of recombinant H3.1 and 500 ng of nucleosomes incubated or not with CREBBP and Pr-CoA) were injected onto a custom-made BioResolve RP (450 Å, 2.7 μm, 0.3 mm × 150 mm) polyphenyl column (Waters) using an Acquity M-Class chromatography system from Waters. Mobile phases A and B were H_2_O and H_2_O/ACN 20/80, respectively, acidified with 0.1% (v/v) difluoroacetic acid. Samples were eluted at a flow rate of 6 μl/min using the following slope change points: 0 to 5 min hold at 12.5% B, 10 min gradient to 25% B, 40 min gradient to 50% B, 10 min wash at 80% B, and finally 5 min hold at 12.5% B. The eluent was sprayed using the conventional electrospray ionization ion source of a Waters Cyclic IMS SELECT SERIES mass spectrometer operating in the positive ion mode. The TOF was operated in V-mode at a mass range of *m/z* 500 to 4000 with the cone voltage set to 80 V. Data were processed using the MassLynx v4.2 software (Waters).

### LC–MS/MS analysis after sample digestion

Samples (corresponding to 1 μg of recombinant H3.1 or nucleosomes incubated with or without CREBBP and Pr-CoA) were diluted with 20 μl of 50 mM NH_4_HCO_3_ buffer containing 0.1 μg of chymotrypsin and incubated overnight at 37 °C. After digestion, 200 ng of these samples were loaded and desalted onto Evotips according to the manufacturer's instruction, prior to LC–MS/MS analysis. Samples were analyzed on a timsTOF Pro 2 mass spectrometer (Bruker Daltonics) coupled to an Evosep one system (Evosep) operating using the 30SPD method developed by the manufacturer. Briefly, the method is based on a 44 min gradient and a total cycle time of 48 min with a C18 analytical column (0.15 × 150 mm, 1.9 μm beads) equilibrated at 40°C and operated at a flow rate of 500 nl/min. H_2_O/0.1% FA was used as solvent A, and ACN/0.1% FA was used as solvent B. The timsTOF Pro 2 was operated in data-dependent acquisition–parallel accumulation serial fragmentation (PASEF) model with a cycle time of 1.3 s. Mass spectra for MS and MS/MS scans were recorded between 100 and 1700 *m/z*. Ion mobility was set to 0.75 to 1.25 V-s/cm^2^ over a ramp time of 180 ms. Data-dependent acquisition was performed using six PASEF MS/MS scans per cycle with a near 100% duty cycle. Low *m/z* and single charged ions were excluded from the PASEF precursor selection by applying a filter in the *m/z* and ion mobility space. Dynamic exclusion was enabled and set to 0.8 min with a target value of 16,000 and an intensity threshold of 1000. Collision energy was incrementally increased as a function of ion mobility. MS raw files were processed using PEAKS Studio 12.5 (Bioinformatics Solutions, Inc). Data were searched against the *Homo sapiens* SwissProt database (downloaded 2022_04; 20376 entries). The parent mass tolerance was set to 20 ppm, and the fragment mass tolerance was set to 0.05 Da. Semi-specific chymotrypsin cleavage was selected, and a maximum of three missed cleavages was allowed. The following post-translational modifications were included for identification purpose: propionyl (K), oxidation (M), and deamidation (NQ) as variables and half of a disulfide bridge (C) as fixed. Identifications were filtered based on a 1% false discovery rate threshold at both the peptide and protein group level, and a minimum A-score of 12 was set, which calculates an ambiguity score as −10 × log10p. The *p* value indicates the probability that the peptide matches by chance.

### Cell culture, transfection, and propionate exposure

CRISPR CREBBP KO HEK293T and RL cells (CREBBP KO RL cells, CREBBP Y1503C RL cells) were cultured in Dulbecco's modified Eagle's medium or RPMI1640 medium, respectively, both supplemented with 10% fetal bovine serum, as previously described (20). Cells were maintained at 37 °C in a humidified incubator with 5% CO_2_. HEK293T cells were transfected with FLAG-CREBBP-WT or FLAG-CREBBP-Y1503C plasmids for 24 h in 100 cm^2^ petri dishes. Transfected HEK293T and RL cells were exposed or not to propionate for 24 h prior to Western blot analysis. Cells were routinely tested for mycoplasma by PCR and 4′,6-diamidino-2-phenylindole staining.

### Cell extracts and acid extraction of endogenous histones

Cells were harvested, washed with PBS, and lysed with lysis buffer (PBS, 1% Triton X-100, protease inhibitors, 3.3 μM trichostatin A, and 20 mM butyrate) on ice for 30 min. The lysates were briefly sonicated (10% power) and centrifuged at 15,600*g* for 15 min at 4 °C. The supernatant was saved (cell extract), and the cell pellet was resuspended in 100 μl 0.2 M HCl and sonicated (10% power) before incubation overnight at 4 °C. The mixture was centrifuged at 15,600*g* for 20 min at 4 °C, and the supernatant (histone extract) was collected and pH-corrected with 1 M Tris. Cell extracts (45 μg) and extracted histones (2 μg) were analyzed by Western blot.

### Crystallization, X-ray diffraction data collection, and structure determination

Crystallization screening trials were done at the crystallography core facility of the Institut Pasteur, Paris (45). The screening was carried out using a Mosquito nanoliter dispensing robot (TPPLabtech), by the sitting drop vapor diffusion method. Briefly, drops were set up by mixing 200 nl of sample (5 mg/ml CREBBP previously incubated with a 5X or 6X molar excess of Pr-CoA or Lys-CoA, respectively) with an equivalent volume of the reservoir solution from the 96-well plates. The plates were stored at 18°C in a RockImager system (Formulatrix), and crystal growth was monitored using an automated imaging system. The best crystals were obtained at 18 °C in wells containing 0.1 M (NH_4_)_2_SO_4_, 30% (w/v) PEG 4000, 0.1 M trisodium citrate, pH 5.6 in the reservoir. Crystals were flash cooled in liquid nitrogen using reservoir solution supplemented with 25% (v/v) ethylene glycol as cryoprotectant. X-ray diffraction data were collected on the PROXIMA-1 beamline at the SOLEIL synchrotron (St Aubin). Crystal structures were determined by molecular replacement method with PHASER (46) using the CREBBP wildtype structure (Protein Data Bank [PDB] ID: 5U7G) as a search model. Model rebuilding and refinement was performed using phenix.refine and Coot (47, 48). Atomic model coordinates and structure factors were deposited in the Research Collaboratory for Structural Bioinformatics PDB under accession codes 9H02 (CREBBP HAT–Pr-CoA complex) and 9H0K (CREBBP HAT–Lys-CoA complex). Structural analyses and pictures were generated using PyMOL (version 2.7.0) (Schrödinger, Inc.).

### Statistical analysis

Data were tested for normal distribution using Shapiro–Wilk test with GraphPad Prism 8.0. Data are presented as means ± SD of three independent experiments and analyzed by two-sided unpaired *t* test using GraphPad Prism 8.0. A threshold of *p* < 0.05 was used to consider differences as statistically significant (∗*p* ≤ 0.05, ∗∗*p* ≤ 0.01).

## Data availability

The coordinates for the two structures reported here have been deposited in the PDB. The accession codes are as follows: CREBBP HAT–Pr-CoA complex (PDB ID: 9H02) and CREBBP HAT–Lys-CoA complex (PDB ID: 9H0K). Plasmids and cell lines used in this study are available upon request. Any further information and request of resources should be directed to and will be fulfilled by F.R.L. (fernando.rodrigues-lima@u-paris.fr).

## Supporting information

This article contains supporting information.

## Conflict of interest

M. R. G. reports research funding from Sanofi, Kite/Gilead, Abbvie, and Allogene; consulting for Abbvie, Allogene, and Bristol Myers Squibb; and Johnson & Johnson and Arvinas. All other authors declare that they have no conflicts of interest with the contents of this article.
