# Supplementary material for: Structural and functional characterization of CREB-binding protein (CREBBP) as a histone propionyltransferase
Source: J Biol Chem. 2025 Jul 2;301(8):110444. doi: 10.1016/j.jbc.2025.110444 (PMC12305233; doi:10.1016/j.jbc.2025.110444)
Supplement: Supplementary Information [file mmc1.docx]

**Supporting information**

**Structural and functional characterization of CREB-binding protein (CREBBP) as a histone propionyltransferase**

Guiling Cui^1^, Marie Ley^2^, Ariel E. Mechaly^3^, Linh-Chi Bui^1^, Christina Michail^1^, Jérémy Berthelet^4^, Julien Dairou^5^, Haopeng Yang^6^, Guillaume Chevreux^2^, Gautier Moroy^1,7^, Michael R. Green^6^, Ahmed Haouz^3^ and Fernando Rodrigues Lima^1,*^

^1^Université Paris Cité, CNRS, Unité de Biologie Fonctionnelle et Adaptative, F-75013 Paris, France

^2^Université Paris Cité, CNRS, Institut Jacques Monod, Plateforme ProtéoSeine, F-75013, Paris, France

^3^Institut Pasteur, CNRS, Plateforme de Cristallographie-C2RT, F-75015, Paris, France

^4^Université Paris Cité, CNRS, Unité Epigénétique et Destin Cellulaire, F-75013, Paris, France

^5^Université Paris Cité, CNRS, Laboratoire de Chimie et de Biochimie Pharmacologiques et Toxicologiques, F-75006, Paris, France

^6^Department of Lymphoma and Myeloma and Department of Genomic Medicine, The University of Texas MD Anderson Cancer Center, Houston, TX, USA

^7^Université Paris Cité, INSERM, Unité de Biologie Fonctionnelle et Adaptative, F-75013 Paris, France.

* Corresponding author: Fernando Rodrigues Lima (fernando.rodrigues-lima@u-paris.fr)

Running title: CREBBP and lysine propionylation


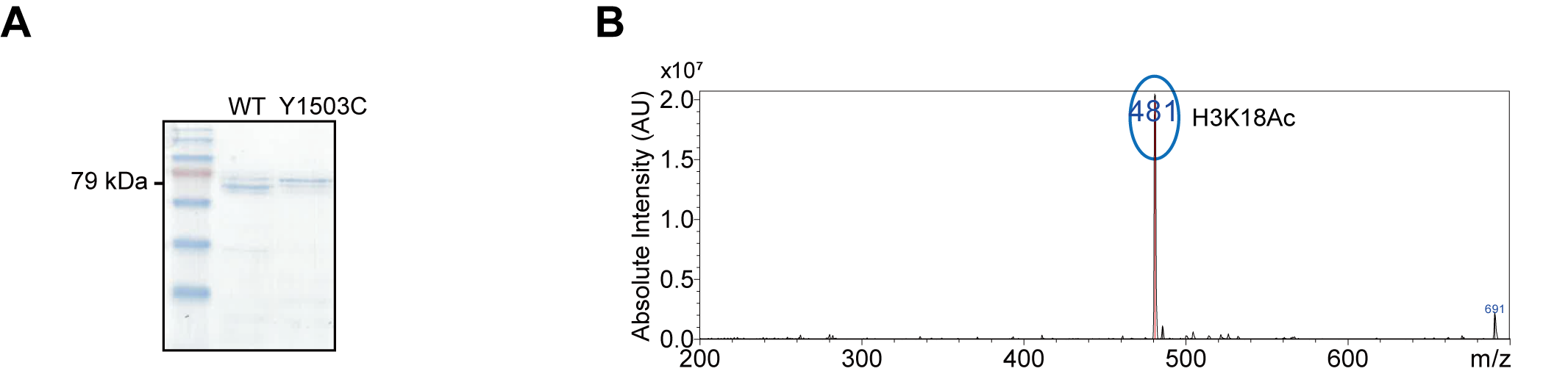


**Supplementary Figure 1**: (**A**) Coomassie staining of recombinant purified CREBBP catalytic core. (**B**) LC-MS spectrum showing acetylation of the H3K18 peptide substrate by CREBBP in the presence of Ac-CoA (m/z ratio equal to 481 Da, corresponding to the triple charged ion calculated as ([M + 3H⁺])/3). The molecular mass of the H3K18Ac peptide is 1439.58 Da.


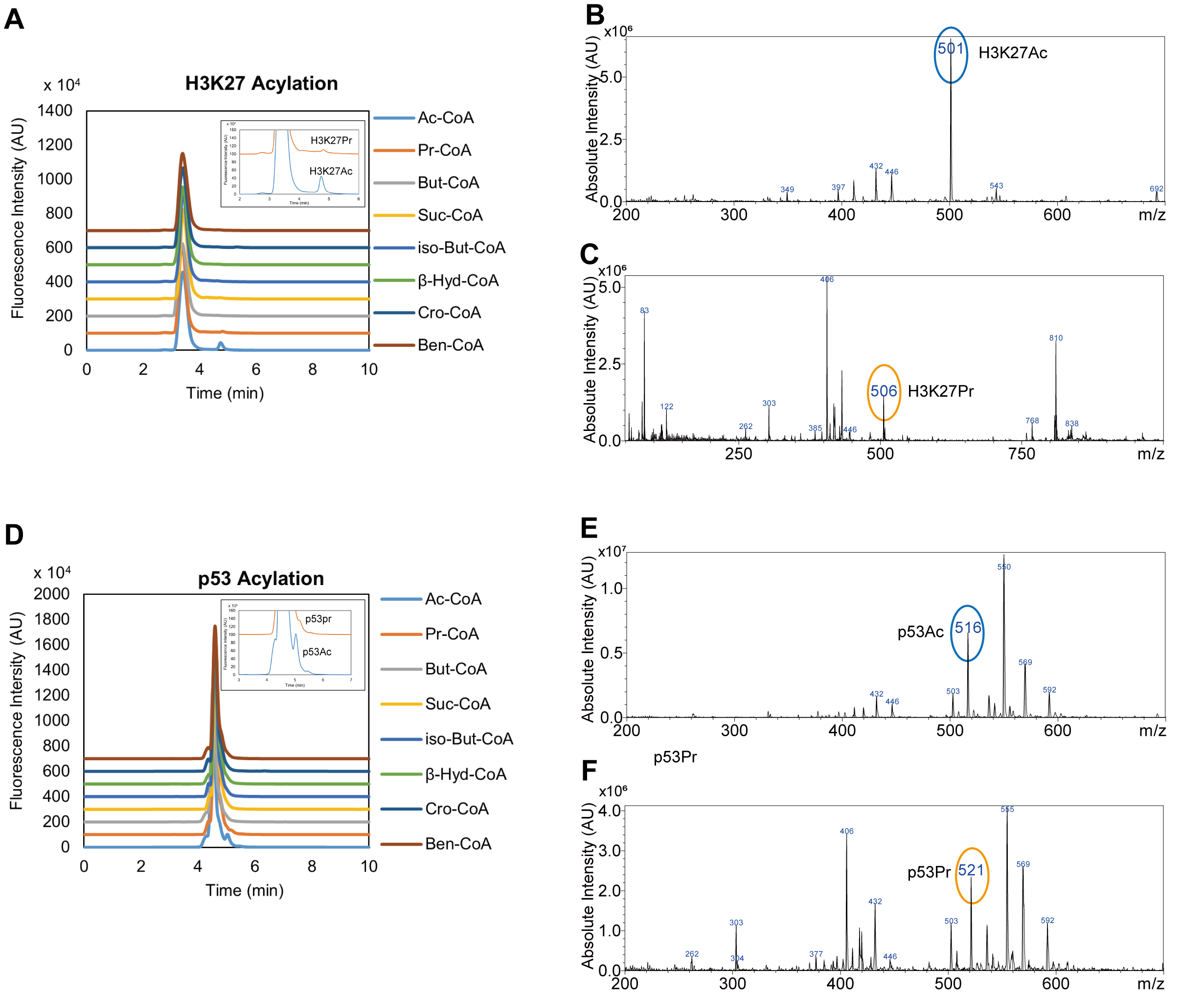


**Supplementary Figure 2:** LC-MS analysis of H3K27 and p53 peptide substrate acylation by CREBBP. **(A)** Chromatogram showing H3K27 peptide acylation by CREBBP in the presence of different acyl-CoAs. **(B)** LC-MS spectrum showing H3K27 acetylation by CREBBP (m/z ratio equal to 501 corresponds to the triple charged ion calculated as ([M + 3H⁺])/3). The molecular mass of the H3K27Ac peptide is 1500.62 Da). **(C)** LC-MS spectrum showing H3K27 propionylation by CREBBP (m/z ratio equal to 506 corresponds to the triple charged ion calculated as ([M + 3H⁺])/3). The molecular mass of the H3K27Pr peptide is 1514.65 Da). (D) Chromatogram showing p53 peptide acylation by CREBBP in the presence of different acyl-CoAs. **(E)** LC-MS spectrum showing p53K382 acetylation by CREBBP (m/z ratio equal to 516 corresponds to the triple charged ion calculated as ([M + 3H⁺])/3). The molecular mass of the p53K382Ac peptide is 1546.78 Da). **(F)** LC-MS spectrum showing p53K382 propionylation by CREBBP (m/z ratio equal to 521 corresponds to the triple charged ion calculated as ([M + 3H⁺])/3). The molecular mass of the p53K382Pr peptide is 1560.81 Da).


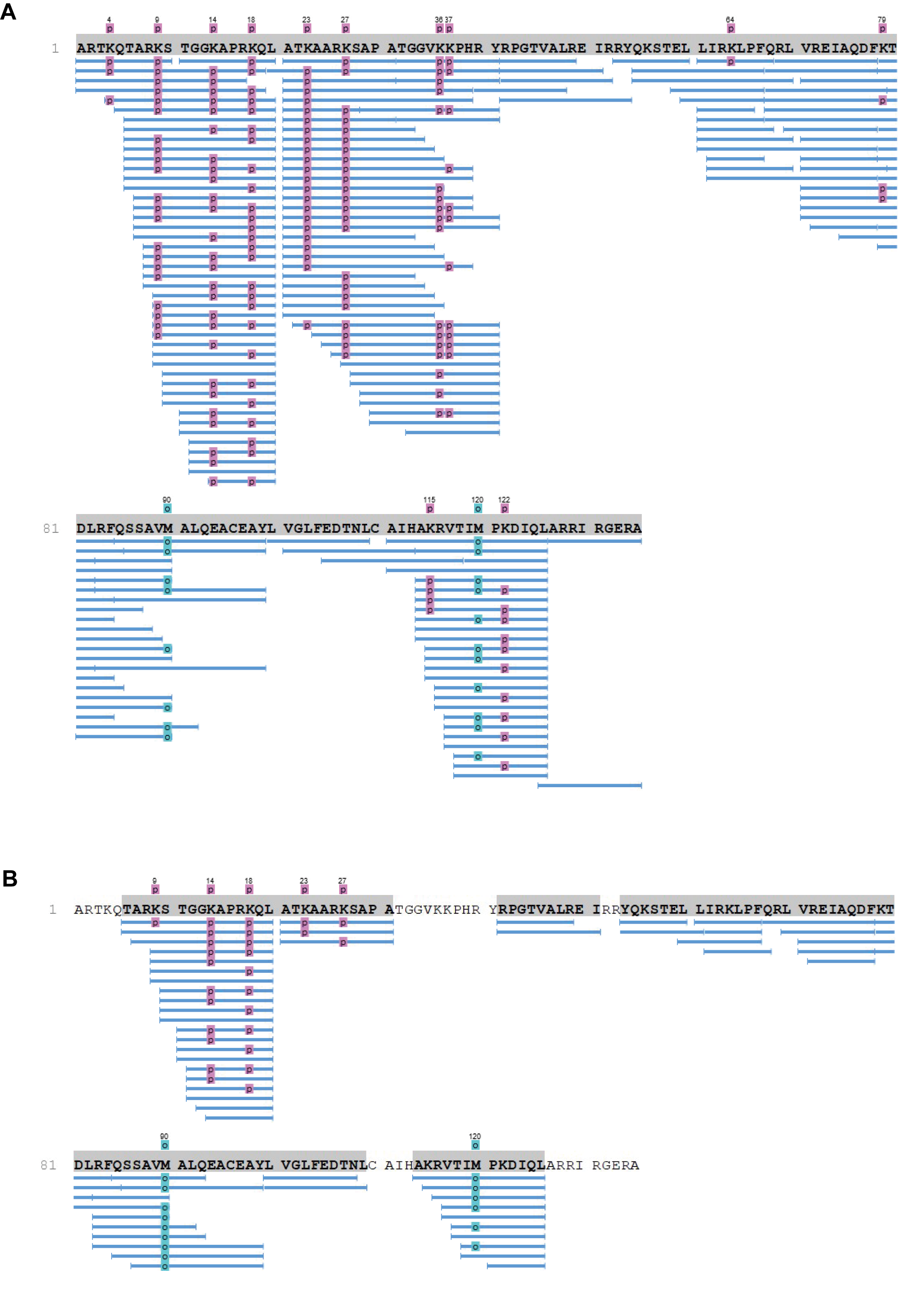


**Supplementary Figure 3:** Coverage map of **(A)** propionylated recombinant H3.1 and **(B)** propionylated H3.1 in nucleosomes. Propionylation sites were annotated in pink and major ones were detected at positions K4, K9, K14, K18, K23, K27, K115 and K122 for recombinant H3.1 and K9, K14, K18, K23, K27 for the H3.1 (nucleosome sample). Oxidized methionines (M90 and M120) are annotated in blue. A minimum A-score of 12 (*p*-value of 0.05) was set corresponding to the localization score assigned to the modifications on the peptide.


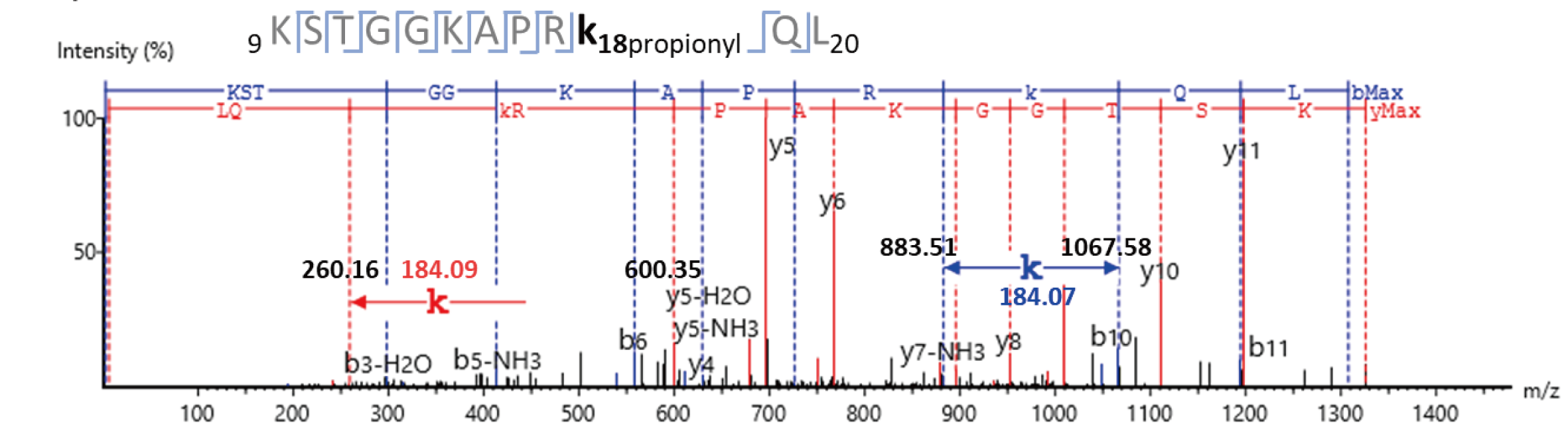


**Supplementary Figure 4:** MS/MS spectrum of peptides showing the propionylation of K18 in recombinant histone H3 by CREBBP (A-score of 46.21) Mass differences supporting evidence of H3K18 propionylation are further annotated on the MS/MS spectrum.


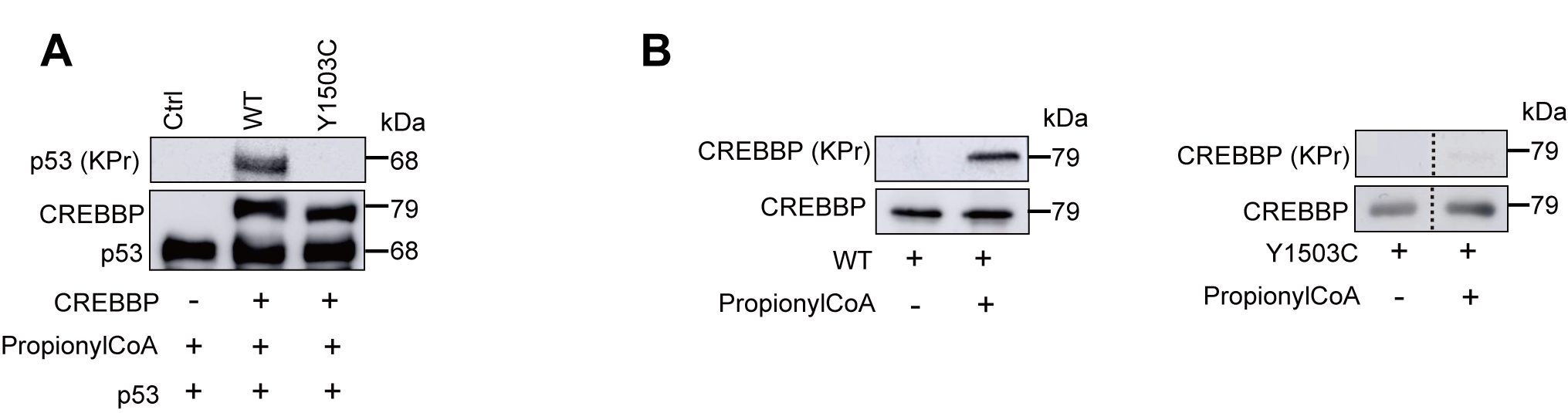


**Supplementary Figure 5:** Western blot analysis showing **(A)** Propionylation of recombinant p53 by CREBBP and **(B)** Auto-propionylation of CREBBP. Lysine propionylation was detected with an anti-pan-propionyl-lysine antibody. Recombinant CREBBP and p53 proteins were detected with an anti-6xHis tag antibody. The inactive CREBBP Y1503C enzyme was used as a control.
